# Supplementary material for: Cyano Enone-Bearing Triterpenoid Soloxolone Methyl Inhibits Epithelial-Mesenchymal Transition of Human Lung Adenocarcinoma Cells In Vitro and Metastasis of Murine Melanoma In Vivo
Source: Molecules. 2020 Dec 14;25(24):5925. doi: 10.3390/molecules25245925 (PMC7765109; doi:10.3390/molecules25245925)
Supplement: Supplementary file 1 [file molecules-25-05925-s001.pdf]

Article

Soloxolone methyl inhibits epithelial-mesenchymal transition of human lung carcinoma cells in vitro and metastasis of murine melanoma in vivo

Andrey V. Markov [1,*,†], Kirill V. Odarenko [1,†], Aleksandra V. Sen'kova [1], Oksana V. Salomatina [1,2], Nariman F. Salakhutdinov [2] and Marina A. Zenkova [1]

[1] Institute of Chemical Biology and Fundamental Medicine, Siberian Branch of the Russian Academy of Sciences, Lavrent'ev ave., 8, 630090 Novosibirsk, Russia; k.odarenko@yandex.ru (K.V.O.); alsenko@mail.ru (A.V.S.); ana@nioch.nsc.ru (O.V.S.); marzen@niboch.nsc.ru (M.A.Z.)

[2] N.N. Vorozhtsov Novosibirsk Institute of Organic Chemistry, Siberian Branch of the Russian Academy of Sciences, Lavrent'ev ave., 9, 630090 Novosibirsk, Russia; anvar@nioch.nsc.ru (N.F.S.)

* Correspondence: andmrkv@gmail.com; Tel.: +7-383-363-51-61 (A.V.M.)

† These authors contributed equally to this work

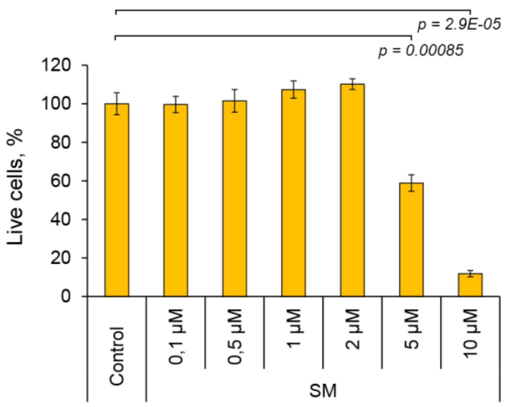

Figure S1. Cytotoxicity of SM against non-malignant human gingival fibroblasts (HGF). HGF were treated by SM at mentioned concentrations for 24 h under standard conditions followed by the measurement of the level of live cells using MTT assay. Error bars represent the standard deviation of two independent experiments performed in tetraplicate.

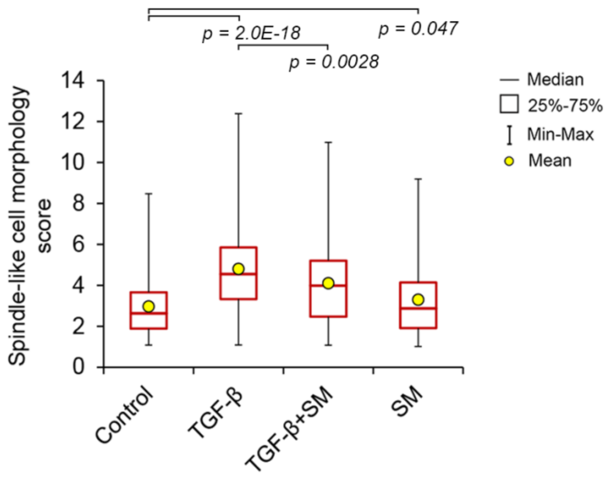

Figure S2. SM suppressed TGF-β-induced stretch of A549 cells. A549 cells were incubated with the presence of TGF-β (50 ng/ml) and/or SM (0.5 μM) for 48 h followed by the evaluation of

cellular morphology by phase contrast microscopy and the calculation of spindle-like cell morphology score using ImageJ tool. Spindle-like cell morphology score was calculated as described in Material and methods (See Section 5.8.).

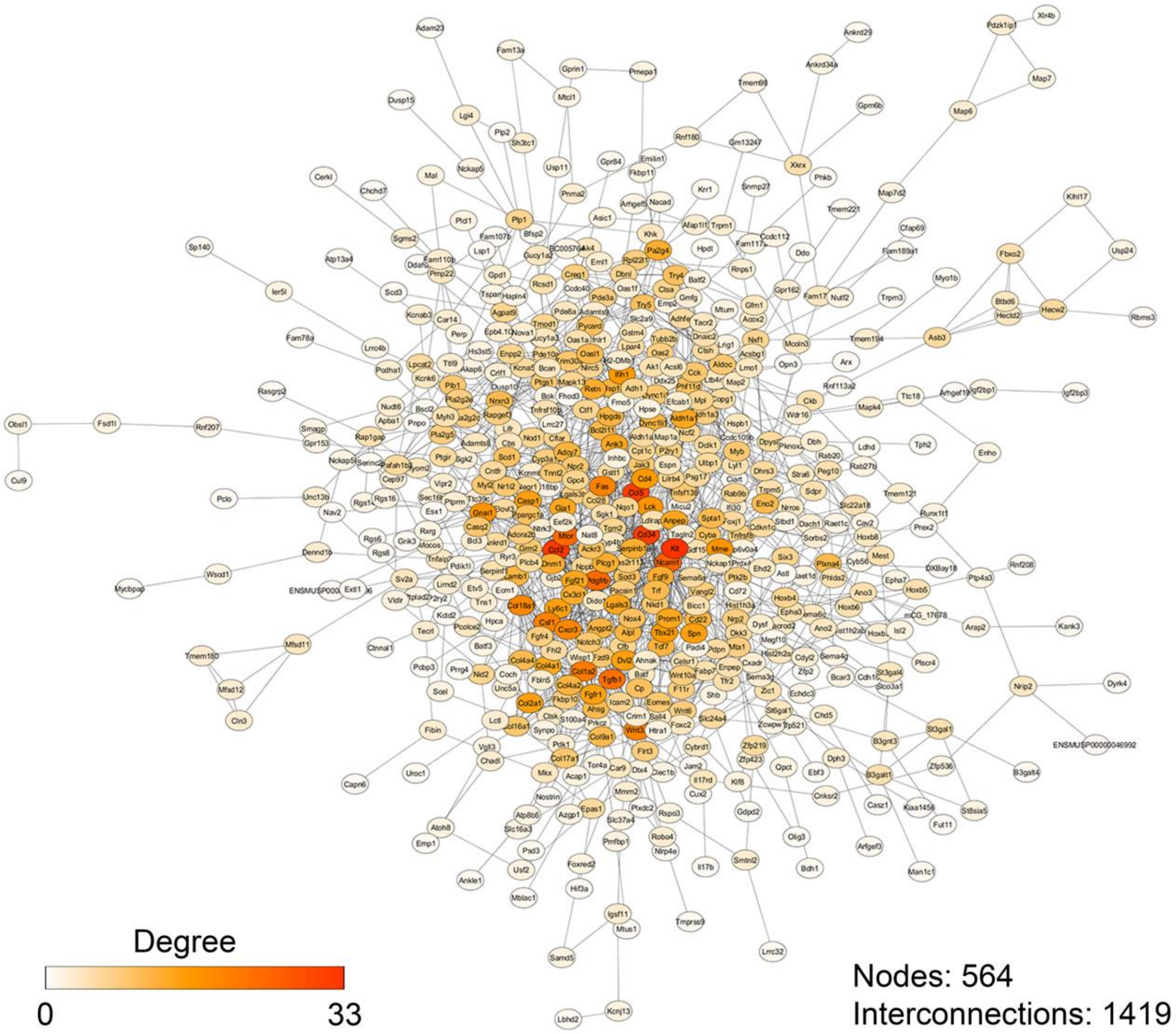

Figure S3. Regulome of melanoma cells associated with highly aggressive phenotype. The gene regulatory network was reconstructed with differentially expressed genes (fold change > 4, $p < 0.05$) revealed after comparison of whole genome expression profiles of aggressive B16 cells vs poorly tumorigenic D5.1G4 melanoma cells (by re-analysis of GSE69908 dataset by GEO2R platform) using STRING database (confidence score > 0.4) and visualized by Cytoscape v. 3.7.2. Only nodes with ≥ 1 interconnections within the network were considered. The ranking of aggressive phenotype-associated DEGs according to their level of interconnection into the network was further carried out using the NetworkAnalyzer plugin.

Table S1. Predicted protein targets of SM

Gene Symbol	Gene name	Source[1]
ADAM17	ADAM Metallopeptidase Domain 17	PPB2
BACE1	Beta-Secretase 1	PPB2
CETP	Cholesteryl Ester Transfer Protein	PPB2

Gene Symbol	Gene name	Source[1]
CNR1	Cannabinoid Receptor 1	PPB2
CNR2	Cannabinoid Receptor 2	PPB2
CYP19A1	Cytochrome P450 Family 19 Subfamily A Member 1	PPB2
CYP2C19	Cytochrome P450 Family 2 Subfamily C Member 19	PPB2
CYP3A4	Cytochrome P450 Family 3 Subfamily A Member 4	PPB2
GPBAR1	G Protein-Coupled Bile Acid Receptor 1	PPB2
KCNH2	Potassium Voltage-Gated Channel Subfamily H Member 2	PPB2
MMP2	Matrix Metalloproteinase-2	PPB2
MMP9	Matrix Metalloproteinase-9	PPB2
MMP13	Matrix Metalloproteinase-13	PPB2
NR3C2	Nuclear Receptor Subfamily 3 Group C Member 2	PPB2
OPRM1	Opioid Receptor Mu 1	PPB2
SLCO1B1	Solute Carrier Organic Anion Transporter Family Member 1B1	PPB2
SLCO1B3	Solute Carrier Organic Anion Transporter Family Member 1B3	PPB2
NOS2	Nitric Oxide Synthase 2	PPB2, STP
HSD11B1	Hydroxysteroid 11-Beta Dehydrogenase 1	PPB2, STP
HSD11B2	Hydroxysteroid 11-Beta Dehydrogenase 2	PPB2, STP
ABCG2	ATP Binding Cassette Subfamily G Member 2	STP
ACACA	Acetyl-CoA Carboxylase Alpha	STP
ACACB	Acetyl-CoA Carboxylase Beta	STP
ADCY10	Adenylate Cyclase 10	STP
ADRA1D	Adrenoceptor Alpha 1D	STP
AR	Androgen Receptor	STP
BRD7	Bromodomain Containing 7	STP
BRD9	Bromodomain Containing 9	STP
CCR3	C-C Motif Chemokine Receptor 3	STP
CCR8	C-C Motif Chemokine Receptor 8	STP
KCNJ1	Potassium Inwardly Rectifying Channel Subfamily J Member 1	STP
MAPK8	Mitogen-Activated Protein Kinase 8 (JNK1)	STP

[1]Probable primary targets of SM were predicted using Polypharmacology Browser PPB2 (PPB2) and SwissTargetPrediction (STP) web services. Top 20 proteins from PPB2 and top 15 proteins from STP were used in further analysis.

Table S2. Sequences of primers for RT-PCR

Gene name	Forward	Reverse
E-cadherin	5′-CCAGAATAAAGACCAAGTGAC-3′	5′-CCAAGAATCCCCAGAATGGCAGGAAT-3′
Vimentin	5′-TGAGATTGCCACCTACAG-3′	5′-TGAGAAGTTTCGTTGATAACC-3′
Fibronectin	5′-CCACTTCCCCTTCCTATAC-3′	5′-TCCCACTGATCTCCAATG-3′
GAPDH	5′-GTGAAGGTCGGAGTCAAC-3′	5′-TGGAATTTGCCATGGGTG-3′
